# Alterations to Melanocortinergic, GABAergic and Cannabinoid Neurotransmission Associated with Olanzapine-Induced Weight Gain

**DOI:** 10.1371/journal.pone.0033548

**Published:** 2012-03-16

**Authors:** Katrina Weston-Green, Xu-Feng Huang, Chao Deng

**Affiliations:** 1 Centre for Translational Neuroscience, School of Health Sciences, University of Wollongong, Wollongong, Australia; 2 Schizophrenia Research Institute, Darlinghurst, Australia; Kaohsiung Chang Gung Memorial Hospital, Taiwan

## Abstract

**Background/Aim:**

Second generation antipsychotics (SGAs) are used to treat schizophrenia but can cause serious metabolic side-effects, such as obesity and diabetes. This study examined the effects of low to high doses of olanzapine on appetite/metabolic regulatory signals in the hypothalamus and brainstem to elucidate the mechanisms underlying olanzapine-induced obesity.

**Methodology/Results:**

Levels of pro-opiomelanocortin (POMC), neuropeptide Y (NPY) and glutamic acid decarboxylase (GAD_65_, enzyme for GABA synthesis) mRNA expression, and cannabinoid CB1 receptor (CB1R) binding density (using [^3^H]SR-141716A) were examined in the arcuate nucleus (Arc) and dorsal vagal complex (DVC) of female Sprague Dawley rats following 0.25, 0.5, 1.0 or 2.0 mg/kg olanzapine or vehicle (3×/day, 14-days). Consistent with its weight gain liability, olanzapine significantly decreased anorexigenic POMC and increased orexigenic NPY mRNA expression in a dose-sensitive manner in the Arc. GAD_65_ mRNA expression increased and CB1R binding density decreased in the Arc and DVC. Alterations to neurotransmission signals in the brain significantly correlated with body weight and adiposity. The minimum dosage threshold required to induce weight gain in the rat was 0.5 mg/kg olanzapine.

**Conclusions:**

Olanzapine-induced weight gain is associated with reduced appetite-inhibiting POMC and increased NPY. This study also supports a role for the CB1R and GABA in the mechanisms underlying weight gain side-effects, possibly by altering POMC transmission. Metabolic dysfunction can be modelled in the female rat using low, clinically-comparable olanzapine doses when administered in-line with the half-life of the drug.

## Introduction

The second generation antipsychotic (SGA) olanzapine is prescribed to treat schizophrenia and a growing number of other disorders in adults and children [1]–[8], but can cause adverse metabolic side-effects including increased body weight [9], caloric intake [10], [11] and abdominal adiposity [12], [13], and reduced physical activity [14]–[16]. Metabolic side-effects are a growing concern due to co-morbidities such as diabetes and obesity [17], and are a risk factor for medication non-compliance [18]. A number of potential mechanisms for SGA-induced metabolic dysfunction have emerged over the past few years [19]–[21]. In particular, the histaminergic, serotonergic and dopaminergic neurotransmitter systems are thought to be highly implicated in SGA-induced body weight gain [21]–[26]. However, SGAs have a broad receptor binding profile that allows direct and indirect effects on multiple neural and peripheral signalling pathways [26], and further research into other candidate systems is required.

The hypothalamic arcuate nucleus (Arc) and the dorsal vagal complex (DVC) of the brainstem are well-documented for their role in appetite and energy homeostasis [27]–[29]; responding to the acute nutritional status and long-term regulation of energy stores in the body. Neurons of the Arc and DVC express G_i/o_-coupled cannabinoid CB1 receptors (CB1R) [30], [31], which facilitate the effects of cannabinoids on appetite and energy metabolism [32]. Weight gain during olanzapine and clozapine treatment is associated with a CB1R gene polymorphism in individuals with chronic schizophrenia [33], and chronic high-dose risperidone treatment increases cannabinoid receptor agonist, [^3^H]CP-55940, binding density in the Arc of male rats [34]. We previously demonstrated a decrease in [^3^H]CP-55940 binding density in the DVC of rats treated with olanzapine, but not aripiprazole or haloperidol [35]. However, whether changes in receptor density were attributed to the CB1R is unclear due to the low specificity of the ligand used [36] and localisation of cannabinoid CB2 receptors in the brain [37]. Moreover, the effects of olanzapine on CB1R density in the Arc remain unknown.

The appetite enhancing effects of the major neuronal inhibitor, γ-aminobutyric acid (GABA) in the hypothalamus were reported more than 30 years ago [38]. GABAergic neurons in the Arc are sensitive to leptin [39], and GABA receptor agonists and antagonists stimulate and suppress feeding behaviour, respectively [40]. Down-regulated expression of glutamic acid decarboxylase (GAD, the GABA synthesising enzyme) has been observed in individuals with schizophrenia, bipolar and mood disorder, whereas antipsychotic drug treatment increases cortical GAD expression in rats and primates [41]. GAD exists as two isoforms, 65 and 67; the latter is found throughout the neuronal cytoplasm, whereas GAD_65_ is located primarily in the axon terminal [42]–[44] and is the predominant transcript in the hypothalamus of the adult rat brain [45]. However, to our knowledge the effects of olanzapine on GAD_65_ mRNA expression in the hypothalamic Arc or the DVC have not been investigated.

The Arc and DVC both express mRNA for orexigenic neuropeptide Y (NPY) and anorexigenic pro-opiomelanocortin (POMC) [46]–[48]. The POMC gene encodes for neuropeptides such as adrenocorticotropic hormone, β-endorphin and α-melanocortin stimulating hormone; the latter of which exerts its anorexigenic effects largely through melanocortin-3 and melanocortin-4 receptor (MC4-R) subtypes [49]. Conversely, the central application of NPY induces food intake in a number of species [50], hypolocomotor activity in rats [51], [52], and can lead to obesity following chronic over-exposure [53], [54]. Therefore, it is possible that interference in the balance of POMC and NPY by olanzapine may contribute to the drug's obesogenic liability. Several reports demonstrated increased NPY immunoreactivity in the Arc of clozapine-treated rats [55], [56], whereas chronic risperidone treatment in male rats had no effect on POMC or NPY expression, or body weight [34], which may be due to the lower sensitivity of male rats to SGA-induced metabolic side-effects compared to females [57]–[60]. Other studies have examined antipsychotic effects on NPY mRNA expression in the brain with region-dependent outcomes [61]–[65]. The effects of antipsychotics on POMC or NPY in the brainstem have not been examined and studies on hypothalamic appetite-regulating peptides during olanzapine treatment are confounding; one group reported an increase in orexigenic NPY and AgRP and a concurrent reduction in appetite-inhibiting POMC and cocaine- and amphetamine-related transcript (CART) [66], whilst another reported no change in several hypothalamic peptides, including NPY and POMC [67]. A key factor that may contribute to the difference in findings is drug dosage (i.e.: 1 mg/kg olanzapine [66] *vs.* a supratherapeutic dose of 3 mg/kg olanzapine [67] (b.i.d.). Indeed, metabolic outcomes can differ with antipsychotic dosage [68]–[71] and increased dose induces greater metabolic dysfunction in the rat [66], [72], [73], however, high antipsychotic dosages in the rat may not represent the clinic [74]. In addition, both studies had a large dosage interval, i.e.: 6–7 then 17–18 hourly treatments, b.i.d. [66], [67]. As the half-life of olanzapine is 5.1 hours in the rat brain with high levels remaining after 8-hours [75], compared to approximately 75.2 hours in the human brain [76], multiple dosages are required in the rat in order to minimise drug fluctuations below sub-therapeutic D2 receptor occupancy levels [74], [77]. Therefore, it may be possible to model olanzapine-induced metabolic dysfunction in the rat using low olanzapine dosages when administered in accordance with the half-life of the drug, i.e.: 8 hourly (t.i.d.) within in 24-hours.

Using an established rat model of olanzapine-induced metabolic dysfunction [35], [70], [78]–[80], this study aimed to investigate the mechanisms underlying weight gain associated with olanzapine treatment by examining its effects on POMC, NPY and GAD_65_ mRNA expression, and CB1R binding density (using the CB1R-specific ligand [^3^H]-SR141716A) in the Arc and DVC. Statistical correlations between these parameters in the brain and body weight, food intake and visceral adiposity were investigated. To identify the minimum dosage threshold required to induce metabolic change, rats were treated with different clinically-relevant olanzapine dosages, calculated based on comparable therapeutic *in-vivo* dopamine D2 receptor occupancy levels [74] and differences in body surface area between species [81]. Collectively, the present study demonstrates that olanzapine changes the balance of anorexigenic POMC and orexigenic NPY mRNA expression in the Arc, does not alter POMC or NPY in the DVC, and increases GAD_65_ mRNA expression but reduces CB1R density in both the hypothalamus and brainstem. These largely dose-sensitive changes may underlie a shift in energy balance that favours weight gain during olanzapine treatment. Metabolic dysfunction can be modelled in the female rat using low olanzapine doses when administered in-line with the half-life of the drug.

## Methods

### Ethics Statement

All experimental procedures were approved by the Animal Ethics Committee (Approval #: AE06/32), University of Wollongong, and complied with the Australian Code of Practice for the Care and Use of Animals for Scientific Purposes (2004). All efforts were made to minimise animal distress and prevent suffering.

### Animals and Treatment

Seven week-old female Sprague Dawley rats (Animal Resources Centre, Perth, WA, Australia), housed in 12-h light–dark cycle (lights on 07:00, 22°C) were habituated for 1-week, then randomly assigned to 0.25, 0.5, 1.0 or 2.0 mg olanzapine/kg (Zyprexa, Eli Lilly, Indianapolis, IN, USA) or vehicle (control) (n = 12), administered three-times daily in a sweet cookie dough pellet, as described previously [70]. Briefly, olanzapine tablets were de-coated and pulverized then the assigned dosage was added to measured dry ingredients. Water droplets were added immediately prior to administration to achieve a dry-dough consistency. After a 1-week teaching period, rats learnt to voluntarily self-administer a 0.3 g cookie-dough pellet either containing the assigned dosage of olanzapine, or plain cookie-dough without the drug (control group), offered by a metal spoon at 8-hourly intervals (3 pellets/day) for 14-days. Consumption of each pellet was observed to ensure complete dosing. Body weight and food intake measurements were recorded (n = 12). Animals were allowed *ad libitum* access to water and standard laboratory chow diet throughout the study. Animals were fasted for 4–6 hours then euthanized using sodium pentobarbitone 10–12 hours after the last treatment. Brain tissue was immediately frozen in liquid nitrogen and stored at −80°C. Visceral (perirenal and periovary) white fat pads were dissected and weighed (n = 12). Six brains were randomly selected from each treatment group for use in the mRNA expression and receptor binding experiments. Tissue was sectioned (14 µm, −18°C) along the coronal plane then stored at −20°C.

### NPY, POMC and GAD_65_ mRNA In-Situ Hybridisation

POMC mRNA expression was observed using in-situ hybridisation techniques previously described by our laboratory [82], using the following specific antisense hybridisation probe: 5′-CGTTCTTGATGATGGCGTTCTTGAAGAGCGTCACCAGGGGCGTCT-3′ (J00612, 547–591). NPY mRNA expression was observed using in-situ hybridisation techniques previously described by our laboratory [65], [82], using the following specific antisense hybridisation probe: 5′-GAGTGTATCTGGCCATGTCCTCTGCTGGCGCGTCCTCGCCCGG-3′ (M15792, 1650–1693). GAD_65_ mRNA expression was observed using the following specific antisense hybridisation probe: 5′-GGCGTCCACACTGCAAGGCCTTGTCTCCCGTGTCATAGGACAGGTCAT-3′ (NM_012563.1, 1419-1372), as previously described by Ling et al., [83]. Oligonucleotide probes were terminally labelled using [^35^S]dATP (1000 Ci/mmol, Perkin Elmer, Waltham, MA, USA) in 10-fold molar excess and terminal transferase (Promega, Madison, WI, USA), then purified using a MicroSpin G-50 column (GE Healthcare Ltd, Buckinghamshire, UK). Hybridisation was performed by incubating slides in hybridisation buffer (4× SSC, 1× Denhardt's solution, 50% de-ionised formamide, 200 µg/ml sperm DNA, 100 µg/1 ml polyA, 120 µg/ml heparin, 20 mM sodium phosphate and labelled probe, pH 7.0) for 18-hours at 37°C. Slides were then washed in 1× SSC buffer at 55°C (3×30-minutes each) and incubated for 1-hour in SSC buffer at room temperature. Sections were dipped in Milli-Q water followed sequentially by 70% then 95% ethanol, and dried under a gentle stream of air. Autoradiographic images were captured on film (Kodak BioMax MR film, Rochester, NY, USA) exposed for 3-weeks. Films were quantified using a GS-800 Densitometer (Bio-Rad Laboratories, Inc), and analysis software (Quantity One, v4.6.7, Bio-Rad Laboratories, Inc, CA, USA). Values were derived from a standard curve generated from a [^14^C]-labelled autoradiographic standard (GE Healthcare Ltd, Buckinghamshire, UK) (mean binding nCi/g tissue equivalent vs. density). Slides were dipped in Emulsion solution (GE Healthcare Ltd, Buckinghamshire, UK) and exposed for 6-weeks, then stained with cresyl violet (Nissl stain) (Sigma-Aldrich, NSW, Australia), to allow further examination of positive signals at the cellular level.

### CB1R Binding Density

CB1R binding density was detected using methods previously published by our laboratory [84]. Briefly, air-dried slides were pre-incubated for 15 min in incubation buffer containing 50 mM Tris–HCl buffer (pH 7.4) and 0.1% bovine serum albumin, at room temperature. Sections were then incubated with 10 nM [^3^H] SR141716A (52 Ci/mMol, Amersham, UK), a CB1R-specific inverse agonist, in buffer (pH 7.4) at room temperature for 60 minutes to determine total binding. Non-specific binding was determined by incubating subsequent sections in 10 nM [^3^H] SR141716A in the presence of 100 µM CP-55940, in buffer (pH 7.4) for 60 minutes at room temperature. Slides were washed in ice cold buffer (pH 7.4), (2×30 minutes), then dipped in distilled water and dried under a gentle stream of cool air. CB1R autoradiographic images were captured using a Beta Image camera (BioSpace, Paris, France), which counts the amount of β-particles emitted from the tissue (3.5 hours exposure) to determine the level of radioactivity bound to the brain sections. Radioactive levels were obtained in counts per minute per square millimetre of tissue (cpm/mm^2^), converted to nCi/mg tissue equivalent using standard tissue sections calibrated with commercial standards (Amersham, Buckinghamshire, United Kingdom), then transformed into fmol/mg tissue equivalent by taking into account the specific activity of the radioligand (52 Ci/mMol). Quantification was conducted using β-Image Plus software (version 4, BioSpace, Paris, France).

### Quantification and Statistical Analysis

Quantification of autoradiographic images was performed on the hypothalamic Arc and the DVC of the brainstem, which were confirmed using a corresponding set of cresyl violet-stained slides and a standard rat brain atlas [85]. Data were analysed using SPSS (version 17.0, SPSS, Chicago, IL, USA). All data points were within ±2 standard deviations. One-Sample Kolmogorov-Smirnov tests revealed normal data distribution. One-way ANOVAs were employed to determine the effect of treatment on percentage body weight change, food intake, visceral adiposity, as well as NPY, POMC and GAD_65_ mRNA expression, and CB1R binding density in the hypothalamus and brainstem. ANOVAs were followed by multiple comparisons using post-hoc Dunnett-T tests where relevant (*p*<0.05). Correlations were identified using Pearson's correlation tests.

## Results

### Body Weight, Food Intake and Visceral Adiposity

**Figure 1:**
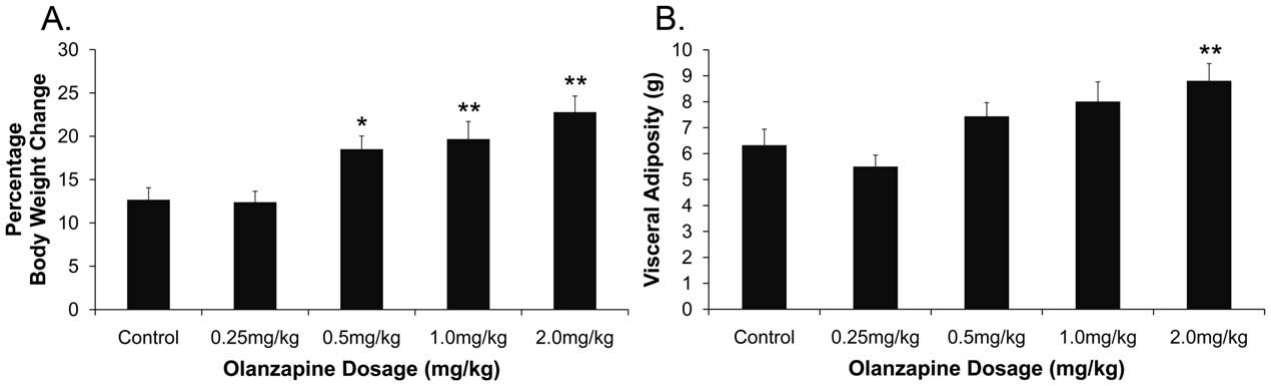
Weight Gain and Visceral Adiposity. (**A**): Percentage body weight change and (**B**): visceral adiposity in female Sprague Dawley rats (*n* = 12/treatment group) treated with 0.25, 0.5, 1.0 or 2.0 mg/kg olanzapine or vehicle (control), (14-days, t.i.d). Data expressed as mean±SEM. *p<0.05, **p<0.01 vs. control.

There was a significant effect of treatment on the percentage of body weight change from treatment day 0 (*F*_4,55_ = 7.68, *p*<0.01). Compared to controls, olanzapine significantly increased percentage of body weight change in the 0.5 mg/kg (*p*<0.05), 1.0 mg/kg and 2.0 mg/kg (*p*<0.01) treatment groups, but not in the low dosage group of 0.25 mg/kg (*p*>0.05) (Figure 1A). Mean cumulative food intake significantly increased in the 2.0 mg/kg olanzapine dosage group compared to the control group (318.6±11.2 g *vs.* 269.4±11.1 g, *p*<0.05). An increase (7–8%) in food intake was also observed in the 0.5 mg/kg and 1.0 mg/kg olanzapine treatment groups, but did not reach significance compared to the controls (0.5 mg/kg: 289.1±13.9 g *vs.* 269.4±11.1 g, 1.0 mg/kg: 291.6±12.1 g *vs.* 269.4±11.1 g, *p*>0.05), and the low dosage group (0.25 mg/kg) did not differ to the control group (265.4±11.7 g *vs.* 269.4±11.1 g). Olanzapine treatment had a significant effect on visceral adiposity (*F*_4,55_ = 4.60, *p*<0.01), with a significant increase observed in the 2.0 mg/kg olanzapine treatment group (*p*<0.05) and a trend for an increase in the 1.0 mg/kg dosage group (*p* = 0.09), but not in the lower dosage groups (*p*>0.05) (Figure 1B).

### POMC and NPY mRNA Expression

**Figure 2:**
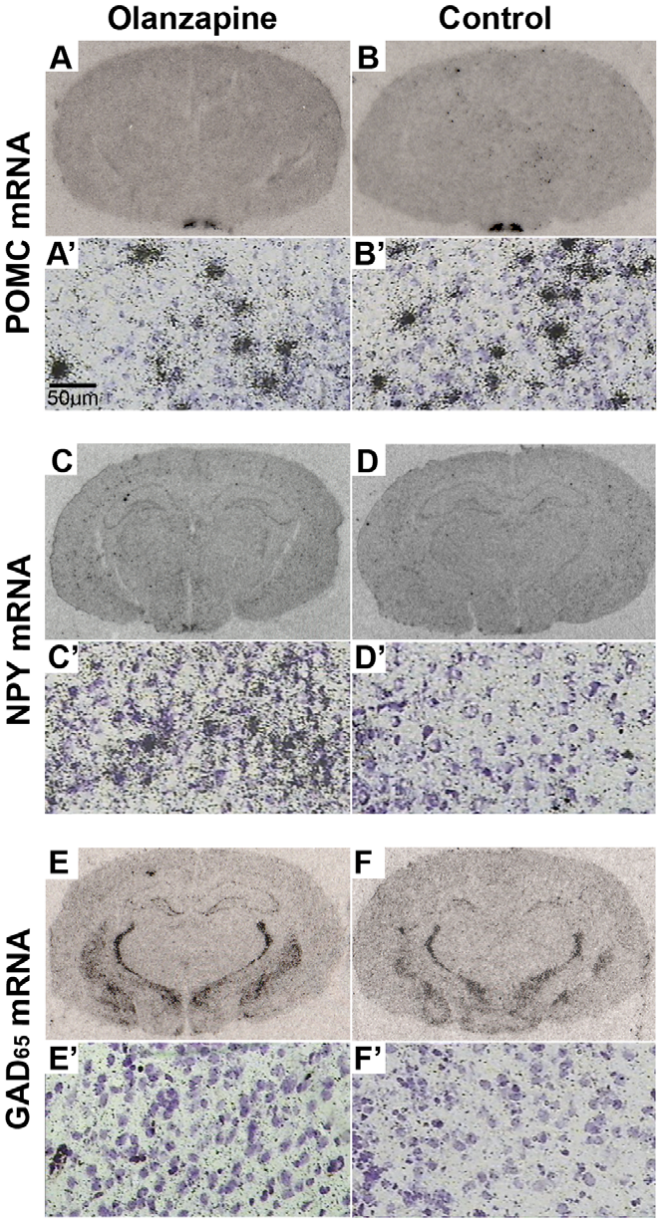
Examples of POMC, NPY and GAD_65_ mRNA Expression Following Olanzapine Treatment. Examples of pro-opiomelanocortin (POMC), neuropeptide Y (NPY) and glutamic acid decarboxylase 65 (GAD_65_) mRNA expression in the female Sprague Dawley rat brain following 2.0 mg/kg olanzapine treatment (**A-A′, C-C′, E-E′**) or vehicle (control) (**B-B′, D-D′, F-F′**) for 14-days (t.i.d.). (**A–F**): Low magnification film autoradiographs depicting mRNA expression in the rat brain, (**A′–F′**): High magnification emulsion/cresyl violet–stained slides showing mRNA expression specifically in the arcuate nucleus. Autoradiographs are examples of raw data used for the graphs depicted in Figure 3A–C and are average representations of 6 rats per treatment group.

**Figure 3:**
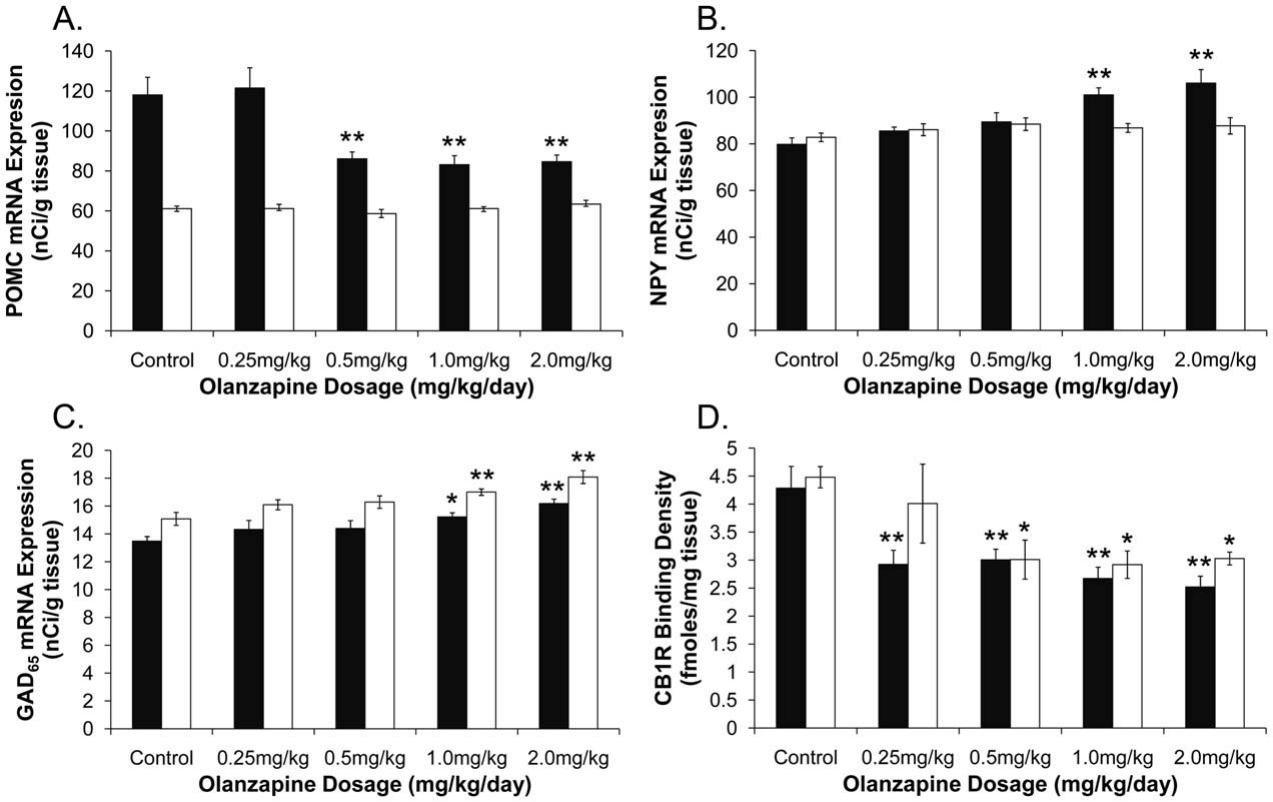
Dosage Effects of Olanzapine Treatment on POMC, NPY and GAD_65_ mRNA Expression, and CB1R Binding Density. (**A**): Pro-opiomelanocortin mRNA expression (nCi/g tissue), (**B**): neuropeptide Y mRNA expression (nCi/g tissue), (**C**): glutamic acid decarboxylase (GAD_65_) mRNA expression (nCi/g tissue), (**D**): cannabinoid CB1 receptor binding density (fmoles/mg tissue), in rats treated with 0.25. 0.5, 1.0 or 2.0 mg/kg olanzapine or vehicle (control) (14-days, t.i.d.) (*n* = 6/treatment group). Key: ▪ arcuate nucleus □ dorsal vagal complex. Data is expressed as mean ± SEM. *p<0.05, **p<0.01 vs. control.

Examples of POMC and NPY mRNA expression in the hypothalamus are shown in Figure 2A–D. Olanzapine had a significant effect on POMC mRNA expression in the Arc (*F*_4,25_ = 8.32, *p*<0.01), not in the DVC (*F*_4,25_ = 1.44, *p* = 0.25) (Figure 3A). Post-hoc analysis identified a significant decrease in POMC mRNA expression in the Arc following dosages of 0.5 mg/kg, 1.0 mg/kg and 2.0 mg/kg (*p*<0.01) olanzapine, but not 0.25 mg/kg olanzapine (*p* = 0.90), compared to controls (Figure 3A). There was also a significant effect of treatment on NPY mRNA expression in the Arc (*F*_4,25_ = 8.55, *p*<0.01), with a significant increase in the Arc following 1.0 mg/kg and 2.0 mg/kg olanzapine (Figure 3B), but not in the lower dosage groups (*p* = 0.34 and *p* = 0.11 for 0.25 mg/kg and 0.5 mg/kg olanzapine, respectively) (Figure 3B). NPY mRNA expression in the DVC was unaltered by olanzapine (*F*_4,25_ = 0.73, *p* = 0.58) (Figure 3B).

### GAD_65_ mRNA Expression

Examples of GAD_65_ mRNA expression are shown in Figure 2E–F. A significant effect of treatment on GAD_65_ mRNA expression was observed in the Arc (*F*_4,25_ = 5.21, *p*<0.01) and DVC (*F*_4,25_ = 7.73, *p*<0.01), with an increase following olanzapine dosages of 1.0 mg/kg (Arc *p*<0.05, DVC *p*<0.01) and 2.0 mg/kg (both regions *p*<0.01), but not in the 0.5 mg/kg or 0.25 mg/kg groups (*p*>0.05), compared to controls (Figure 3C).

### CB1R Binding Density

**Figure 4:**
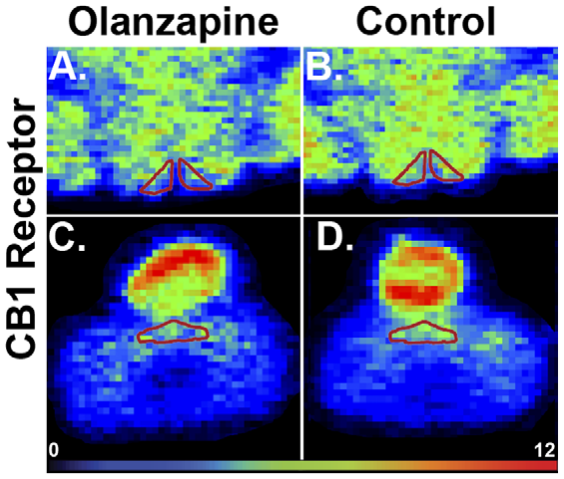
Example of CB1R Binding Density Following Olanzapine Treatment. Example of cannabinoid CB1 receptor binding density (using [^3^H]SR-141716A) in the **A, C:** hypothalamic arcuate nucleus and **B, D:** dorsal vagal complex of the caudal brainstem of female Sprague Dawley rats following **A, B:** 2.0 mg/kg olanzapine treatment, **C, D:** vehicle (control) for 14-days (t.i.d.). Autoradiographs are examples of raw data used for the graphs depicted in Figure 3D and are average representations of 6 rats per treatment group.

An example of CB1R binding density in the hypothalamus and DVC is shown in Figure 4. There was a significant effect of treatment on CB1R binding density in the Arc (*F*_4,25_ = 7.48, *p*<0.01), with a reduction in all olanzapine treatment groups compared to controls (*p*<0.01) (Figure 3D). Olanzapine decreased CB1R binding density in the DVC (*F*_4,25_ = 3.48, *p*<0.05) of animals treated with 0.5 mg/kg, 1.0 mg/kg or 2.0 mg/kg olanzapine (*p*<0.05), but not 0.25 mg/kg olanzapine (*p* = 0.43) (Figure 3D).

### Correlations

**Figure 5:**
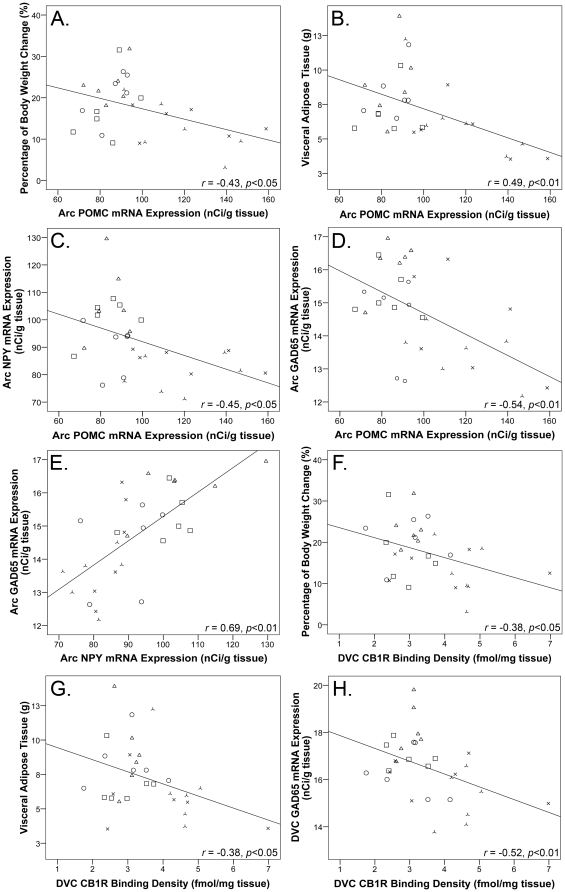
Correlations. Correlations between pro-opiomelanocortin (POMC) mRNA expression in the arcuate (Arc) nucleus and (**A**): percentage of body weight change, (**B**): visceral adipose tissue, (**C**): neuropeptide Y (NPY) mRNA expression in the Arc, (**D**): glutamic acid decarboxylase (GAD_65_) mRNA expression in the Arc, (**E**): Arc NPY and GAD_65_ mRNA expression, (**F**): cannabinoid CB1 receptor (CB1R) binding density in the dorsal vagal complex (DVC) and percentage of body weight changed, (**G**): DVC CB1R binding density and visceral adipose tissue, and (**H**): DVC CB1R binding density and GAD_65_ mRNA expression, following 14-days olanzapine treatment or vehicle (control). Correlation analyses were made from raw data underlying the graphs presented in Figures 1 and 3. Key: <$>\raster="rg1"<$> control, ×0.5 mg/kg, ○ 0.5 mg/kg, □ 1.0 mg/kg, ▵ 2.0 mg/kg olanzapine.

POMC mRNA expression in the Arc significantly correlated to percentage body weight change (*r* = −0.43, *p*<0.05), visceral adiposity (*r* = −0.49, *p*<0.01), NPY mRNA expression (*r* = −0.45, *p*<0.05), and GAD_65_ mRNA expression in the Arc (*r* = −0.54, *p*<0.01) (Figure 5A–D). There was a significant positive correlation between NPY and GAD_65_ mRNA expression in the Arc (*r* = 0.69, *p*<0.01) (Figure 5E), however the two factors did not correlate to percentage body weight change (*p*>0.05). CB1R binding density in the DVC correlated to percentage body weight change (*r* = −0.38, *p*<0.05), visceral adiposity (*r* = −0.38, *p*<0.05) and GAD_65_ mRNA expression in the DVC (*r* = −0.52, *p*<0.01) (Figure 5F–H), and a weak correlation was observed between CB1R binding density in the Arc and percentage body weight change (*r* = −0.33, *p* = 0.08).

## Discussion

We found that olanzapine alters signals in the hypothalamus and brainstem that are implicated in appetite and energy homeostasis in a largely dose-sensitive manner. These changes may underlie a shift in energy balance that favours weight gain. The data support a role for POMC in the mechanisms underlying olanzapine-induced obesity. Reduced POMC satiety signalling leads to obesity in the clinic and in animal models of obesity, for example, POMC mRNA expression is attenuated in genetically obese Zucker rats [86], tubby mice (*tub* gene mutation) [87] and diet-induced obese mice [88]. In addition, genetic POMC deficiency leads to obesity in humans [89] and mice [90], and MC4-R deficiency leads to morbid obesity associated with enhanced adiposity and chronic hyperphagia [91]. The result of unaltered POMC mRNA expression in the DVC was not entirely surprising as the role of POMC neurons in the DVC is not well-characterised, and functional and chemical distinctions to the Arc have been identified [92], [93].

NPY mRNA expression was upregulated in the Arc following 1.0 mg/kg and 2.0 mg/kg olanzapine treatment, however no significant correlation with weight gain was observed. This is consistent with some NPY transgenic and deficiency models i.e.: mice and rats that over-express NPY do not have a hyperphagic/obese phenotype [94], and genetic modelling of NPY-deficiency does not result in reduced body weight, adiposity, or food intake [95]–[97]. However, it is possible that NPY had an indirect effect on weight gain in olanzapine-treated animals, for example by inhibiting POMC. Indeed, NPY neurons synapse on POMC cell bodies and can inhibit their spontaneous activity [98]–[100], however, unlike POMC, NPY mRNA expression did not change in the 0.5 mg/kg olanzapine treatment group suggesting a role for other systems in POMC regulation. The dosage response of NPY mRNA expression was in-line with the increase in GAD_65_ mRNA expression in the 1.0 mg/kg and 2.0 mg/kg olanzapine treatment groups, although GAD_65_ mRNA expression increased in both the Arc and DVC. Upregulated GABAergic signalling during weight gain is consistent with previous reports that whilst NPY and/or AgRP gene deficiency is insufficient to reduce food intake [101], ablation of NPY/AgRP/GABA neurons results in acute hypophagia [102] and deletion of vesicular GABA transporter in AgRP neurons (which co-express NPY) results in a lean, obesity-resistant phenotype in mice [103]. GABA is co-localised in approximately 30% of POMC [104] and NPY/AgRP neurons in the Arc [105], and GABA derived from NPY/GABA axons can suppress spontaneous firing of POMC neurons [98]. In addition, a dense population of leptin-responsive, largely non-AgRP GABAergic neurons that increase inhibitory post-synaptic currents in POMC neurons were recently identified in the Arc [39]. Therefore, the increase in Arc GAD mRNA expression observed in the present study may have arisen from a number of GABAergic sources.

**Figure 6:**
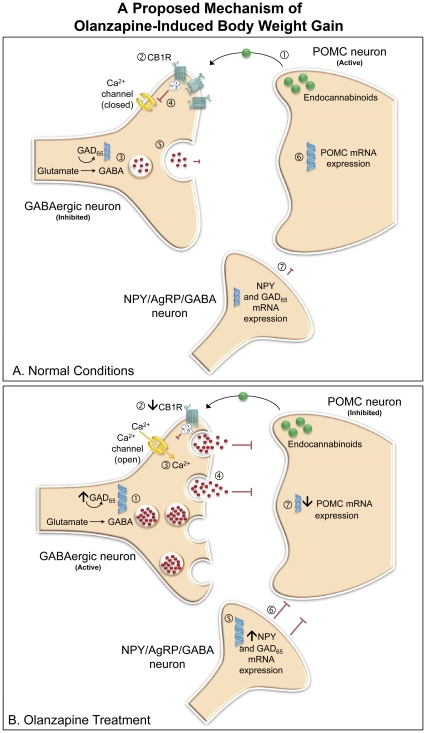
A Proposed Mechanism for Olanzapine-Induced Weight Gain through Interactions Between POMC, NPY, CB1 and GABA systems. (**A**) **Normal Conditions.** Schematic illustrating proposed inhibitory synaptic transmission to the POMC neuron modulated by NPY, cannabinoid and GABAergic systems under normal conditions. (1) Endogenous cannabinoids are released from the post-synaptic POMC neuron and retrogradely activate CB1 receptors located on the non-AgRP GABAergic neuron (2). GABA is synthesised from glutamate via the rate limiting enzyme GAD (3), however G-protein sub-units coupled to the CB1R inhibit the opening of calcium channels (4), which reduces vesicular release of GABA from the presynaptic terminal to the POMC neuron (5), disinhibiting POMC (6). A number of NPY neurons co-express GABA and can also inhibit POMC. These NPY/AgRP/GABA neurons synapse on POMC neurons and can regulate POMC cell activity (7). (**B**) **Olanzapine Treatment**: Our data demonstrates that olanzapine decreases POMC mRNA expression and CB1R binding density, whilst simultaneously increasing NPY and GAD mRNA expression. Based on these findings we suggest a potential mechanism contributing to weight gain during olanzapine treatment. (1) Increased GAD mRNA expression enhances the potential for GABA production, whilst (2) decreased CB1R density following olanzapine treatment may remove inhibition of calcium channels (3) and allow vesicular release of GABA (4). The combined effect may be to increase GABA production and release. (5) Olanzapine increases NPY mRNA expression, which can inhibit POMC activation (6). Therefore, reduced CB1R density, and enhanced GAD and NPY may contribute to the suppression of POMC (7). As POMC is an important anorexigenic peptide, its prolonged inhibition during olanzapine treatment may lead to increased body weight and adiposity side-effects.

Olanzapine treatment elicited a robust reduction in CB1R density in the Arc and DVC. Using the CB1R-specific ligand, [^3^H]-SR141716A, we confirm that our original findings of a reduction in [^3^H]CP-55940 binding density in the DVC during olanzapine treatment [35] were attributed to the CB1R sub-type and extend these findings into the hypothalamus. CB1R number and cell signal transduction pathways decrease following over-exposure to agonists [106] and animal models of obesity, for example obese *db/db* and *ob/ob* mice, and fatty Zucker rats, exhibit elevated hypothalamic endocannabinoid levels [107]. Therefore, reduced CB1R binding density following olanzapine treatment may be a result of increased endogenous cannabinoids. Endogenous cannabinoids play an important regulatory role in synaptic transmission by modulating neuronal excitatory and inhibitory input [108], [109]. Interestingly, POMC neurons secrete endocannabinoids under basal conditions that retrogradely activate CB1Rs expressed on GABAergic neurons [110], [111]. G-protein sub-units coupled to the CB1R inhibit the opening of calcium channels, which reduces vesicular release of GABA [109]. CB1R activation can relieve inhibitory input to the post-synaptic POMC neuron [110]–[112]. We suggest that reduced CB1R density during olanzapine treatment may diminish cannabinoid-regulated inhibition of GABA, and therefore enhance GABAergic input to POMC neurons, suppressing POMC and encouraging body weight gain (Figure 6). In addition, anandamide and CP-55940 increase NPY release in the hypothalamus [113], therefore, increased endocannabinoid levels may contribute to an increase in NPY during olanzapine treatment that further suppresses POMC (Figure 6). CB1Rs can also modulate GABA and glutamate release in the DVC [30], however the functional implications of changes in CB1R density and GAD_65_ mRNA expression during olanzapine treatment require further investigation. Additionally, the influence of olanzapine on other neurotransmitter systems may play a role in the mechanisms underlying SGA-induced weight gain [25], [114]. For example, olanzapine is a potent histamine H1 receptor antagonist [115] and antipsychotic affinity for the H1 receptor can predict its weight gain liability [114], however the underlying mechanisms for the effect of the H1 receptor on antipsychotic-induced body weight may be independent of melanocortinergic neurotransmission [116]. On the other hand, dopamine D1 and D2 receptor antagonism influences hypothalamic NPY mRNA expression [117]–[119] and serotonin 5-HT_2C_ receptor agonists can activate POMC neurons [120], [121], therefore, the antagonistic affinity of olanzapine to D2 and 5-HT_2C_ receptors [122], [123] may contribute to its weight gain side-effects [24]. These receptors may form broader components of the mechanism proposed in the present study, however further research is necessary.

Our finding of a decrease in POMC and increase in NPY mRNA expression during olanzapine treatment coincide with Ferno et al [66], but contrast to the lack of change reported by Davoodi et al [67]. As discussed earlier, these studies differ in olanzapine dosage and treatment interval [66], [67]. Additionally, in Davoodi's study animals were not fasted and PCR methods were used to detect expressional changes in the whole hypothalamus [67], whereas rats were fasted prior to euthanasia and *in-situ* hybridisation techniques were utilised to target expression specifically in the Arc in the present study and [66]. Furthermore, patterns of daily changes in hypothalamic NPY and POMC gene expression have been reported [124], therefore timing of euthanasia may also confound results. A previous study from our laboratory reported a drug withdrawal response of NPY mRNA expression to olanzapine treatment cessation, i.e.: no change in Arc NPY mRNA expression after 2-hour drug washout and a decrease after 48-hour withdrawal after 5-weeks olanzapine treatment [65]. As body weight associated with olanzapine treatment follows a ‘peak-and-plateau’ trend over time [71], [79], [125], the lack of change in NPY mRNA expression [65] may be related to compensatory mechanisms that coincide with a plateau in body weight. Further investigation into the time-dependent pattern of NPY mRNA expression during chronic olanzapine treatment would be useful.

Secher et al. [34] reported increased [^3^H]CP-55940 binding density in the Arc following 28-days risperidone treatment, and observed a significant correlation between plasma drug levels and visceral adiposity. These results are similar to our study as olanzapine influenced CB1R density in the Arc and changes in CB1R density correlated with adiposity. Differences in the direction of CB1R density change may be attributed to several differences in experimental design in Secher et al.'s study [34], including drug dosage above the upper clinical limit [34], administration method i.e.: continuous drug application via mini-pump with no drug washout period, and treatment duration as time-dependent changes in CB1R density and transduction pathways have been reported [106]. Neither drugs have an affinity for the CB1R (>10,000 K_i_ (nM) [126], [127]), indicating that effects on the CB1R are secondary changes and exactly how these SGAs influence CB1Rs should be investigated in future studies. An olanzapine-induced decrease in CB1R binding density seems contrary to the orexigenic influence of CB1R activation, and appetite suppression of CB1R blockade [128]. However, there is vast potential for the endogenous cannabinoid system to modulate metabolism, including central and peripheral effects on food intake and reward aspects of feeding, glucose and lipid metabolism, and energy expenditure [129]–[136]; aspects of which may contribute to the weight loss efficacy of rimonabant [135]. Olanzapine-induced weight gain is associated with increased GABA and decreased CB1R density, whereas anorexigenic rimonabant, decreases GABA release from NPY/AgRP/GABA neurons, possibly via modulation of cannabinoid-sensitive opioid peptides [112]. This suggests that although olanzapine and rimonabant influence the CB1R, they exert their effects through different modes of action.

The doses used in the present study were selected based on the recommended clinical olanzapine dosage range of 5–20 mg/day [70], [74], [81] excluding the 0.25 mg/kg treatment group, which was included as a minimum response threshold. Olanzapine was administered every 8-hours, based on the half-life of olanzapine in the rat brain [75], to minimise inappropriate peaks and troughs in drug levels between treatments [74]. The present study demonstrates that olanzapine-induced metabolic dysfunction can be modelled in the female rat using low olanzapine dosages when treatment is administered in accordance with the half-life of the drug. In addition, treatment was voluntarily self-administered orally in a cookie-dough pellet, which aimed to minimise potential handling stress [137] and maintain a consistently high drug dosage in the brain [74], [75]. Oral drug administration in rats requires a teaching period to ensure voluntary pellet consumption, however this method resembles clinical administration and may circumvent limitations reported using other administration techniques, such as mini-pump, injection and gavage [74], [138]–[140]. Consistent with the clinic [141], olanzapine has a sedative effect in the rat at high doses [72] and we previously reported a general trend of reduced locomotor activity in response to increasing olanzapine dosage [70]. It is possible that sedation plays a role in weight gain during olanzapine treatment, however, as hyperphagia was only apparent in the high dosage group (2 mg/kg olanzapine) in the present study, it is unlikely that sedation influenced the animal's ability to consume food.

In conclusion, our data demonstrates that olanzapine, an antipsychotic drug with a high metabolic liability, alters key metabolic signals in the hypothalamus and brainstem in a manner that favours positive energy balance and may contribute to its weight gain/obesity side-effects. Olanzapine decreases anorexigenic POMC, increases orexigenic NPY, and alters CB1R and GABAergic signalling in a largely dose-sensitive manner. Low dosages of 0.5 mg/kg and 1.0 mg/kg olanzapine (t.i.d.) were sufficient to induce metabolic changes. Drug dosage may contribute, in-part, to inconsistencies observed between reports in the literature. Enhanced body weight and visceral adiposity during olanzapine treatment are associated with reduced anorexigenic POMC mRNA expression. We propose that increased NPY and enhanced inhibitory GABAergic input, possibly through reduced CB1R density, may contribute to POMC inhibition (Figure 6). However, the present study has several limitations, firstly, statistical correlations do not provide direct evidence of a causal link, and secondly, changes to mRNA and receptor binding density may not reflect a functional protein change, therefore further studies are required to confirm the mechanism proposed in the present study. Examination of olanzapine's effects on the GAD_67_ isoform and other hypothalamic neuropeptides, such as AgRP and CART, would be useful, as well as investigation into the time-response of all parameters at different intervals during treatment. Finally, as CB1R density decreased in all olanzapine dosage groups, experiments using lower dosages are required to identify the minimum dosage threshold. Taken together, this study supports a role for the melanocortinergic, GABAergic and cannabinoid systems in the underlying mechanisms contributing to olanzapine-induced weight gain side-effects and provides direction on dosage consideration for future animal modelling studies.
